# Digital Impressions Versus Conventional Impressions in Prosthodontics: A Systematic Review

**DOI:** 10.7759/cureus.51537

**Published:** 2024-01-02

**Authors:** Suhael Ahmed, Abeer Hawsah, Randa Rustom, Abeer Alamri, Sameer Althomairy, Maha Alenezi, Sarah Shaker, Faisal Alrawsaa, Ahmed Althumairy, Abdullah Alteraigi

**Affiliations:** 1 Maxillofacial Surgery, College of Medicine and Dentistry, Riyadh Elm University, Riyadh, SAU; 2 Dentistry, Ministry of Health Saudi Arabia, Riyadh, SAU; 3 Dentistry, Semmelweis University, Budapest, HUN; 4 Dentistry, College of Dentistry, Riyadh Elm University, Riyadh, SAU; 5 Dentistry, Ain Shams General Hospital, Cairo, EGY; 6 Dentistry, Majmaah University, Majmaah, SAU

**Keywords:** prosthodontics, dentistry, dental implants, dental impression, impression accuracy, dental impression technique, digital dentistry

## Abstract

The accuracy of definitive impressions has a significant impact on the quality of the final prosthesis. Elastic impression materials are commonly used in the traditional approach to replicate anatomical structures while indirectly fabricating prostheses. Digital impression has gained increasing popularity due to its various advantages, including three-dimensional previsualization, cost-effectiveness, and reduced time consumption. The objective of this study is to evaluate existing studies to provide an overview of the comparative advantages of digital impression techniques over conventional techniques. The review will focus on evaluating the accuracy, patient acceptability, operator preference, and time effectiveness of digital impression techniques in comparison to conventional techniques. The Population, Intervention, Comparison, and Outcome framework served as the basis for this study’s search strategy. We conducted a comprehensive literature review by electronically searching articles published between 2000 and 2023 in PubMed, Medline, Cochrane, and the Web of Science. Furthermore, additional manual searches were conducted. The study examined the differences between optical impressions and traditional impressions in terms of accuracy, patient outcomes, and operator outcomes. It included both clinical and preclinical studies as well as randomized controlled trials. In conclusion, this review provides a short summary indicating that digital impressions exhibit comparable accuracy to conventional impressions without any statistically significant difference. This conclusion is based on an evaluation of accuracy, patient preference, and operator preference.

## Introduction and background

The use of digital impressions for fabricating crowns, bridges, and dentures has revolutionized the field of prosthodontics by providing a more efficient and accurate method for capturing intraoral data. In the late 1980s, the concept of digital impressions was introduced, but it was not until the early 2000s that the technology became more widely available and commercially viable. Initially, digital scanners were bulky and expensive, limiting their accessibility to a few pioneering dental practices [1,2]. However, with advancements in technology and the development of smaller, more affordable scanners, digital impression systems have become increasingly prevalent in dental offices worldwide.

Advantages of digital impressions include increased accuracy and precision compared to traditional impressions, reduced patient discomfort and gag reflex due to the elimination of messy impression materials, faster turnaround time for restorations as digital impressions can be instantly transmitted to dental laboratories, improved communication between dentists and dental technicians through the ability to share digital files and collaborate remotely, and enhanced patient education and treatment planning with the ability to visualize and manipulate digital models [3]. Overall, digital dentistry offers numerous benefits and advancements in the field of dentistry. It has revolutionized the way dental professionals work, allowing for more efficient and effective treatments. From improved communication to cost savings, digital dentistry has significantly enhanced the patient experience and outcomes [4]. With its integration with other digital technologies, dentists can now provide seamless and precise dental procedures, resulting in better oral health for patients. Digital dentistry is undoubtedly shaping the future of dental care, making it more accessible and convenient for everyone involved.

## Review

Methodology

We conducted a systematic literature review by searching three electronic databases, namely, PubMed, Medline, Cochrane, and Web of Science, for papers published between 2000 and 2023. The search strategy employed a hybrid approach, incorporating both controlled vocabulary and free-text terms. The subsequent section presents the comprehensive search strategy and tactics, which encompassed the selection of keywords devised for each database.

**Figure 1:**
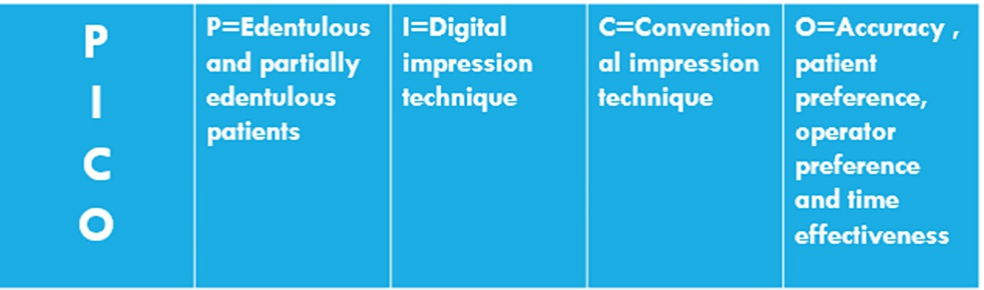
Population, Intervention, Comparison, and Outcome (PICO) framework.

This review was performed following the PICO approach (Patient or Population, Intervention, Comparison, Outcome, and Study types) (Figure 1). The PICO study framework was used to form the following search strategy: P = edentulous and partially edentulous patients; I = digital impression technique; C = conventional impression technique; O = accuracy, patient preference, operator preference, and time effectiveness.

Inclusion and Exclusion Criteria

The study encompassed articles published in the English language within the domain of dentistry in peer-reviewed dental journals. We thoroughly examined the reference lists of articles comparing digital impressions to conventional impressions to uncover further investigations. Papers that remained unpublished, abstracts or case reports that were not published, and reports that solely focused on either conventional or digital impression procedures were excluded from the analysis. We then searched for abstracts to accompany the process of selecting titles for inclusion. After agreeing to include abstracts, we conducted a comprehensive search of full-text articles. The ultimate determination of the articles included in the study was predicated upon a comprehensive examination of the complete text. The study structured the research methodology as follows: the initial step was conducting an electronic search using the keywords “digital impression in prosthodontics.” After conducting the electronic search using the keywords “digital impression in prosthodontics,” we reviewed the abstracts and comprehensively examined the full texts. We finally undertook a careful selection process to identify the final articles for inclusion.

Two reviewers, SA and NT, conducted a separate search of the existing literature to identify and choose relevant titles and abstracts. The two investigators effectively handled the issue of inclusion through deliberation, resulting in the resolution of any disagreements. If the title and abstract did not provide sufficient information regarding the inclusion criteria, the investigators analyzed the entire report. Two reviewers conducted a comprehensive assessment of relevant papers and extracted data separately. The data were obtained by utilizing data extraction tables. The two reviewers successfully handled the issue of data extraction through a collaborative conversation.

Results

**Figure 2:**
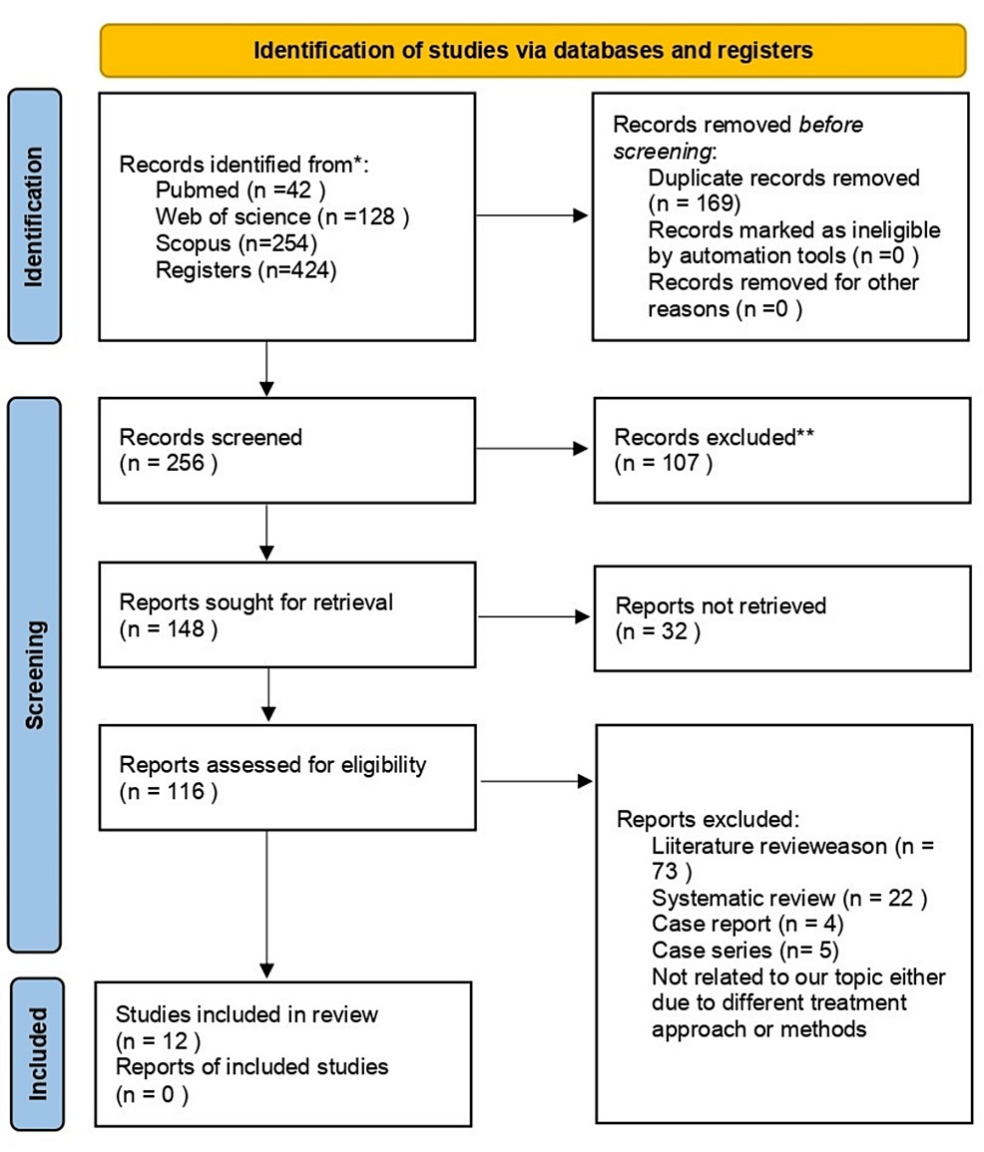
Preferred Reporting Items for Systematic Reviews and Meta-Analyses 2020 flow diagram.

The search resulted in a total of 638 articles from three databases on the subject. Short-listed articles resulted in the retrieval of 48 articles from the PubMed database, 29 from the Web of Science database, and eight from the Cochrane Central Register of Controlled Trials (Figure 2). After evaluating the titles, abstracts, and complete texts, we selected a total of 16 publications from PubMed, nine from Web of Science, and one from the Cochrane Central Register of Controlled Trials. Identifying duplicate studies across two databases reduced the overall number of articles to 12.

Assessment of Risk of Bias

Assessing the methodological rigor of published research studies provides insight into the reliability and validity of the evidence presented. However, it is possible that no singular method for evaluating the methodological validity of systematic reviews is applicable. Therefore, contextual, pragmatic, and methodological factors are taken into account when evaluating the content of studies. The modified Risk of Bias (ROB) 2 assessment was utilized in this instance.

**Figure 3:**
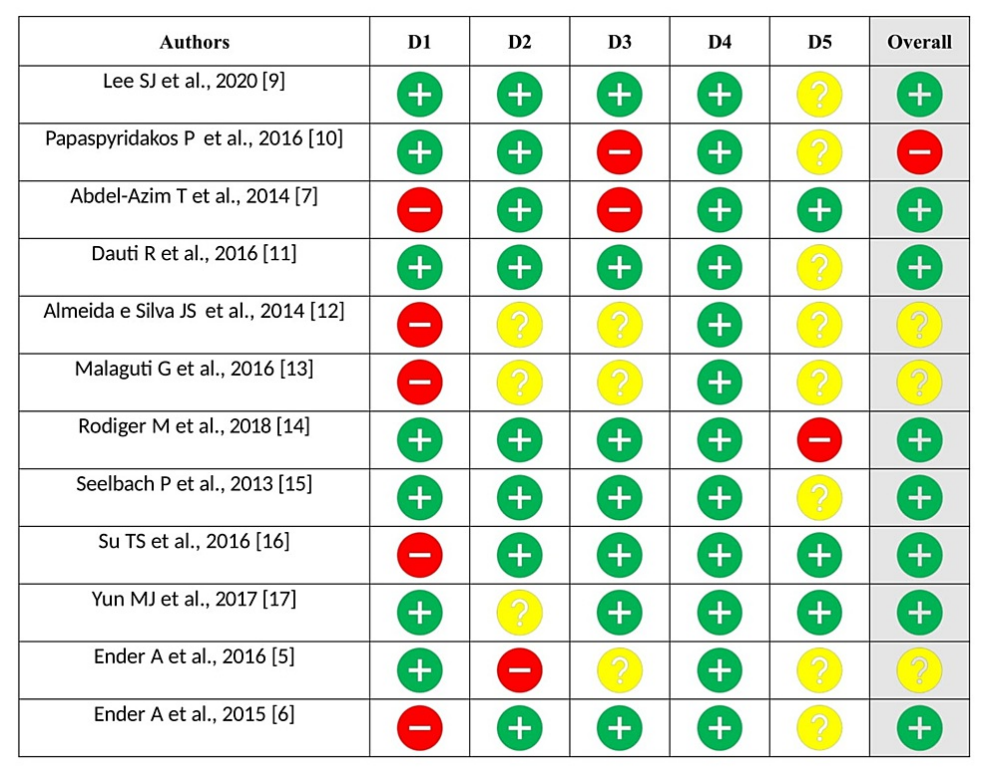
Risk of bias assessment with the recommended approach of Cochrane ROB 2. D1: Bias resulting from the randomization process. D2: Bias resulting from a departure from the intended interventions. D3: Bias resulting from missing data outcomes. D4: Bias resulting from faulty measurement of the outcome. D5: Bias resulting from the selective reporting of results. The risk of bias is indicated by the colors red (high), yellow (some), and green (low).

Figure 3 details the five bias domains that comprise ROB 2. The domains were chosen to encompass all significant mechanisms through which bias may infiltrate trial outcomes. This decision was made in light of empirical evidence and theoretical deliberations. Domains for features that are anticipated to operate indirectly via the included bias domains were omitted from the analysis. Due to this rationale, we omitted certain trial characteristics, including funding source and the distinction between single-center and multicenter status, which have been empirically linked to trial effect estimates.

Discussion

All investigations consistently reported that the marginal gaps observed in crowns created using digital impressions consistently met the criteria for being considered clinically acceptable [5]. Our review found that all marginal gaps were below the threshold of 120 µm, which is considered clinically acceptable. There was a notable disparity in terms of accuracy and precision between digital impression systems and traditional impression processes. Inaccurate fitting might arise in big prosthetic restorations due to local errors above 100 µm, resulting in complications [6,7].

The precision of the impression and the suitability of the final prosthesis are contingent upon each stage of the procedure. To achieve optimal fit precision in conventional methodologies, each step, starting with impressions, stone castings, wax patterns, investment, and casting, must be executed with the utmost care and attention to detail. On the other hand, dental computer-aided design (CAD)/computer-aided manufacturing (CAM) systems generally necessitate a diminished quantity of procedural phases, specifically digital impression, design, and milling. Consequently, the conventional method is associated with a greater number of potential error sources compared to dental CAD/CAM systems [8,9].

Furthermore, the implementation of a standardized milling procedure is crucial. Digital impressions resulted in greater local variations during the fabrication of full-arch fixed dental prostheses compared to conventional impressions. The findings of this study indicate that CAD/CAM systems and digital impressions demonstrate an equivalent level of accuracy to traditional impressions, which has therapeutic implications. Digital impression technologies exhibit superior time efficiency compared to conventional processes [10,11]. Doctors with limited experience find digital impressions more accessible from an operator perspective. Conversely, intraoral cameras face difficulties in effectively capturing distal objectives.

The dimensions of digital intraoral cameras continue to exceed those of conventional imprint trays. Certain digital systems, such as CEREC Bluecam, necessitate the utilization of titanium oxide to enhance contrast. Dental digital imprint systems have a comparatively reduced quantity of error sources compared to conventional impression procedures. The hardware display screen facilitates the monitoring of digital impressions, allowing for the seamless reproduction of inadequately scanned items while retaining the entirety of the impression data. Intraoral cameras are often considered a more comfortable and less intrusive alternative for individuals with a sensitive gag reflex or excessive salivation. Additionally, the transmission of data through these cameras is both cost-effective and expeditious. Additionally, the storage of digital impression data is more convenient. The integration of digital technology has become widely prevalent in various specialized domains of dentistry, including radiography. Conversely, the presence of substantial investment expenditures serves as a hindrance to the adoption and implementation of various technologies.

Evaluation of the Accuracy and Reliability of Digital Impressions Compared to Traditional Impressions

Many studies have shown that digital impressions can produce comparable or even superior results to conventional methods in terms of precision [12-14]. However, it is crucial to acknowledge that there are certain limitations and challenges associated with digital impressions. One of the main challenges is the initial cost of acquiring the necessary equipment and software for digital impressions. This investment can be significant for dental practices, especially smaller ones. Additionally, there is a learning curve for dentists to become proficient in using the new technology. Training and practice are necessary to ensure accurate and efficient digital impressions.

Ender and Mehl conducted an in vitro study to evaluate the accuracy of full-arch dental impressions using traditional and digital imprint procedures. The study utilized four digital imprint technologies, namely, CEREC Bluecam, CEREC AC Omnicam, iTero, and Lava C.O.S., in addition to four traditional impression materials. This study utilized a very accurate reference scanner to evaluate the precision of full-arch conventional impressions versus digital impressions that captured the same tooth morphology. The results showed that CEREC Bluecam, vinylsiloxanether, and direct scannable vinylsiloxanether had the highest levels of accuracy and precision. Generally, digital impression technologies showed more significant regional differences in the whole-arch imprint compared to traditional impression methods. Sealbach and colleagues conducted an in vivo study to evaluate the feasibility and precision of digital scanning. A total of 10 complete-arch intraoral scans were obtained utilizing the iTero CAD/CAM technology, in addition to 10 traditional impregnum impressions, all derived from a single patient. The findings indicated that the patient’s iTero scans exhibited the lowest precision [15].

Ender and Mehl aimed to evaluate the efficacy of a new reference scanner in measuring the accuracy of both conventional and digital full-arch imprints. This study aimed to assess the accuracy and consistency of five traditional impressions using a vinylsiloxanether imprint material as well as five digital impressions created using the CEREC AC Omnica system utilizing an in vitro approach. The results revealed that the digital complete-arch imprint had lower accuracy compared to the traditional impression in both trueness and precision [5].

Researchers observed different deviation patterns in traditional and digital impressions. Ender and Meh conducted an in vitro study to evaluate the precision of traditional and digital imprints in full-arch scanning. The study’s findings revealed that the accuracy of digital impressions was equivalent to that of traditional impressions [6]. Abdel-Azim et al. performed a laboratory study to assess how computer-aided impression alternatives affect the accuracy of dental implant-based single units and complete-arch frameworks. The researchers observed that conventional impressions resulted in a lower level of disagreement compared to digital approaches when considering a single-implant framework. Anadioti et al. conducted an in vitro investigation of crowns in both three-dimensional (3D) and two-dimensional (2D) measurements. The manufacture of crowns entailed the employment of both digital and traditional impressions. The measurements revealed that the group using the polyvinyl siloxane impression/IPS e.max press had the best level of accuracy in terms of marginal fit. The study aimed to examine the marginal inaccuracy and internal fit of the crowns. The study determined that the accuracy of crowns manufactured through the use of digital impressions was equivalent to that of crowns made using traditional impressions [7,8]. Table 1 depicts the summary of selected articles.

**Table 1:** Summary of selected articles.

Study/Specimen ID	Parameter compared	Scan device and software	Accuracy measurement	Main outcome
Lee et al., 2020 [9]	Precision	Cadent iTero TM ; i-Tero	The STL dataset and scanned impression data combined via superimposition	Acceptable values showed by both
Papaspyridakos et al., 2016 [10]	Precision	3 shape; TRIOS	The STL dataset and scanned impression data combined via superimposition	Acceptable values showed by both
Abdel-Azim et al., 2014 [7]	Marginal fit	iTero (Cadent) and Lava COS (3M ESPE),	Stereomicroscope	Digital impression is superior to conventional
Dauti et al., 2016 [11]	Marginal fit	Lava cos	Scanning electron microscope and optical microscope	Acceptable values showed by both
Almeida e Silva et al., 2014 [12]	Internal fit and marginal fit	Lava COS (3M ESPE)	Microscopic examination and computer software	Digital impression is superior to conventional
Malaguti et al., 2016 [13]	Marginal gap and internal gap	Intraoral scanner-MHT scanner 3D progress, extraoral scanner-dental wing series 7	The STL dataset and scanned impression data combined via superimposition	Digital impression is superior to conventional
Rodiger et al., 2018 [14]	Internal fit and marginal fit	TRIOS system	A camera integrated with light a microscope	Acceptable values showed by both
Seelbach et al., 2013 [15]	Internal fit and marginal fit	Lava C.O.S., CEREC AC, and iTero	3D-coordinate measuring system, with a traveling microscope	Digital impression is superior to conventional
Su et al., 2016 [16]	Internal fit and marginal fit	Trios cart	Stereomicroscopy	Digital impression is superior to conventional
Yun et al., 2017 [17]	Internal fit and marginal fit	iTero	Replica method and measuring microscope	Digital impression is superior to conventional
Ender et al., 2016 [5]	Precision	CEREC Omnicam (OC; Sirona Dental Systems), True Definition Scanner (T-Def; 3M ESPE), 3 Shape Trios (TRI; 3 Shape), Cadent iTero (ITE; Cadten Ltd), Lava COS (LAV; 3M ESPE)	Overlaying utilizing specialized diagnostic software	Conventional impression is superior to digital
Ender et al., 2015 [6]	Precision	Cadten Ltd) Lava COS (LAV; 3M ESPE)CEREC Omnicam (OC; Sirona Dental Systems) Cadent iTero (ITE; Cadten Ltd	The STL dataset and scanned impression data combined via superimposition	Digital impression is superior to conventional

Cost-Effectiveness Analysis of Digital Impressions

While the initial investment may seem high, digital impressions can ultimately save dental practices money in the long run. This is because digital impressions eliminate the need for traditional impression materials, such as alginate or polyvinyl siloxane, which can be expensive and require frequent replenishment. In addition, digital impressions can decrease the number of patient visits by easily sharing digital files with dental laboratories for restoration fabrication, eliminating the need to ship physical models back and forth. This not only saves time but also reduces shipping costs and the risk of damage to the physical models during transportation. Additionally, digital impressions provide a more accurate representation of the patient’s oral anatomy, resulting in better-fitting restorations and improved patient satisfaction [16]. With digital impressions, dentists can also easily store and access patient records, allowing for efficient communication and treatment planning. Overall, the adoption of digital impressions in dentistry offers numerous benefits in terms of cost-effectiveness, convenience, accuracy, and patient care.

Patient Satisfaction and Comfort Levels With Digital Impressions

Studies have shown that digital impressions can lead to higher patient satisfaction due to the elimination of uncomfortable traditional impression materials [17]. Patients often find the process of making digital impressions to be quicker and more convenient, resulting in a more positive overall experience. Overall, embracing digital impressions can significantly enhance the dental experience for both practitioners and patients.

Time Efficiency and Operator Perception

Numerous studies have compared conventional and digital impressions, considering perspectives from both the patient and the dentist. A study by Yuzbasioglu et al. (2014) provided evidence that the implementation of digital impression techniques led to a decrease in both the total treatment time and the time required to create an impression in comparison to traditional approaches. The duration of the digital impression was measured by researchers to be 248.48 ± 23.48 seconds, whereas the traditional impression required 605.38 ± 23.66 seconds. The patients expressed a greater degree of satisfaction when digital impressions were taken using the CEREC AC Omnicam system [18]. As a result, they chose this method of treatment.

The digital imprint approach provides enhanced efficiency and convenience in comparison to the conventional impression method, as evidenced by an in vivo study. Superior occlusal contacts have been produced through the implementation of digital technology as opposed to conventional impression techniques. It was determined that the mean duration of procedures for digital and conventional imprint methods was 14 minutes and 33 seconds, with a standard deviation of 5 minutes and 27 seconds, and 20 minutes and 42 seconds, with the same mean duration and a standard deviation of 5 minutes and 42 seconds, respectively. In a similar vein, the mean impression durations for traditional and digital methods were 7 minutes and 33 seconds and 11 minutes and 33 seconds, respectively, with standard deviations of 3 minutes and 37 seconds and 1 minute and 56 seconds. The mean scores obtained by dentists on a Visual Analog Scale (VAS) ranging from 0 to 100 for the digital and conventional imprint techniques were 24.00 ± 18.02 and 48.02 ± 21.21, respectively, in terms of difficulty. The mean values for patients’ assessments of distress on the VAS were 6.50 ± 5.87 for digital imprint procedures and 44.86 ± 27.13 for conventional imprint procedures. A score of 100 hundred on the VAS scale denotes the most severe degree of discomfort [19].

Challenges and Limitations of Digital Impressions

Addressing limited accessibility in certain dental practices or regions is another challenge. The infrastructural constraints or lack of financial means by some dental practices to acquire digital impression systems may restrict the availability of this technology in particular regions or communities. Furthermore, certain practices may be unable to afford the expenses associated with implementing and maintaining digital impression systems, which exacerbates the problem of restricted accessibility. Nevertheless, endeavors are underway to enhance the accessibility of digital dentistry. This includes the provision of training and support to practices that express interest in implementing this technology, as well as the introduction of financing alternatives. With the advancement of accessibility, an increasing number of dental professionals will have the capacity to profit from digital impressions. Notwithstanding these obstacles, digital impressions will persist in providing advantages for dental practitioners, as they are indisputable and anticipated to bring about a paradigm shift in the discipline in the forthcoming years.

## Conclusions

Digital impressions and CAD/CAM have changed prosthodontics. Virtual treatment planning and simulation employing digital impressions and 3D imaging have increased denture production accuracy and predictability. Digital impressions have many advantages over conventional impression procedures, they improve patient as well as operator comfort, reduce the number of visits, and improve the practice efficiency of the operator.
